# Molecular Characterization and Drought Resistance of GmNAC3 Transcription Factor in *Glycine max* (L.) Merr.

**DOI:** 10.3390/ijms232012378

**Published:** 2022-10-16

**Authors:** Zhanyu Chen, Xiaoqin Yang, Minghao Tang, Yujue Wang, Qian Zhang, Huiying Li, Ying Zhou, Fengjie Sun, Xiyan Cui

**Affiliations:** 1 College of Life Sciences, Jilin Agricultural University, Changchun 130118, China; 2 College of Agronomy, Jilin Agricultural University, Changchun 130118, China; 3 Department of Biological Sciences, School of Science and Technology, Georgia Gwinnett College, Lawrenceville, GA 30043, USA

**Keywords:** *Glycine max*, GmNAC3 transcription factor, drought resistance, *Arabidopsis*, PEG6000, superoxide dismutase, catalase, peroxidase, proline, malondialdehyde

## Abstract

Soybean transcription factor GmNAC plays important roles in plant resistance to environmental stresses. In this study, *GmNAC3* was cloned in the drought tolerant soybean variety “Jiyu47”, with the molecular properties of GmNAC3 characterized to establish its candidacy as a NAC transcription factor. The yeast self-activation experiments revealed the transcriptional activation activity of GmNAC3, which was localized in the nucleus by the subcellular localization analysis. The highest expression of *GmNAC3* was detected in roots in the podding stage of soybean, and in roots of soybean seedlings treated with 20% PEG6000 for 12 h, which was 16 times higher compared with the control. In the transgenic soybean hairy roots obtained by the *Agrobacterium*-mediated method treated with 20% PEG6000 for 12 h, the activities of superoxide dismutase, peroxidase, and catalase and the content of proline were increased, the malondialdehyde content was decreased, and the expressions of stress resistance-related genes (i.e., *APX2*, *LEA14*, *6PGDH*, and *P5CS*) were up-regulated. These expression patterns were confirmed by transgenic *Arabidopsis thaliana* with the overexpression of *GmNAC3*. This study provided strong scientific evidence to support further investigation of the regulatory function of *GmNAC3* in plant drought resistance and the molecular mechanisms regulating the plant response to environmental stresses.

## 1. Introduction

Soybean is an important oil and protein crop worldwide, while environmental stresses such as drought and high salt severely affect the growth, yield, and quality of soybean. Therefore, the study of the stress resistance or tolerance of soybean is of significant importance in molecular breeding of soybean varieties [1,2,3,4]. Studies have shown that transcription factors bind indirectly or directly to the functional element region of the promoter to mainly regulate the expressions of downstream genes, and play important roles in plant response to abiotic stresses [5]. For example, numerous members of a large number of transcription factor families (e.g., NAC, bHLH, MYB, WRKY, and AREB) regulate the expressions of plant stress resistance-related genes [6,7,8]. As one of the largest families of plant-specific transcription factors [9], the NAC transcription factors play important roles in plant development, senescence, formation of secondary cell walls, and biotic and abiotic stress response [9,10,11,12,13]. Since its first discovery in 1996 [14], numerous genes of NAC transcription factors have been identified in various plant species, such as 117 genes in *Arabidopsis thaliana*, 151 in rice, 152 in soybean, 180 in apple, 167 in banana, and 177 in sea buckthorn [15,16,17,18,19,20].

The molecular structure and functions of the transcription factors in the NAC family have been well characterized [21,22], significantly facilitating the identification and further characterization of novel NAC transcription factors. For example, studies showed that most of the total of 152 NAC transcription factors identified in soybean were hydrophilic proteins with the negative average hydrophobicity of all amino acids [23]. Furthermore, trivial variations were revealed in the amino acid sequences in the NAC domains based on a total of 75 predicted NAC proteins from rice and 105 NAC genes in *A. thaliana* genome [24], while many stress response-related *NAC* genes were identified in the SNAC group based on the 151 non-redundant *NAC* genes in rice and 117 genes in *A. thaliana* [25,26]. It was demonstrated that the AtNAM transcription factors in *A. thaliana* showed self-activation activities in a yeast system [27]. Moreover, the characterization of the molecular structure of eight *NAC* genes in rice (i.e., *OsNAC1* to *OsNAC8*) revealed their regulatory functions in the growth and development of rice [28].

The molecular functions of the NAC family of transcription factors in regulating the plant response to drought stress have been widely investigated. For example, numerous studies have shown that many genes encoding the NAC transcription factors are involved in the response to drought stress, such as *ATF1*, *ANACO16*, and *ANAC019* in *A. thaliana* [29,30,31], *SNAC3*, *ONACO22*, and *OsNAC14* in rice [32,33,34], *TaNAC29*, *TaNAC47*, and *TaSNAC8-6A* in wheat [35,36,37], *AhNAC3* in peanut [38], and *ZmNAC55* and *ZmNAC48* in maize [39,40]. The overexpression of these genes could significantly improve drought tolerance in transgenic plants. For example, a drought resistance gene *TaSNAC8-6A* identified in wheat was closely related to the drought tolerance of wheat seedlings [37], while the overexpression of *MdNAC1* in apple plants significantly promoted photosynthesis and the activity of active oxygen scavenging enzymes, ultimately improving the drought tolerance of apple plants [41]. Furthermore, the increased drought tolerance in transgenic soybean plants with overexpression of *GmNAC8*, in comparison with that of the wild type and the plants with knockout of *GmNAC8*, suggested that *GmNAC8* played a positive regulatory function in drought response [42]. Moreover, under drought stress, the enzymatic activities of superoxide dismutase (SOD) and the proline content in the soybean lines with the overexpression of *GmNAC* were significantly higher than those in the wild type, while the SOD activity and proline content in the soybean plants with the *GmNAC8* knockout were significantly lower than those in the wild type [42]. The overexpression of the *ONAC066* gene in rice significantly improved the drought tolerance and oxidation, increased the contents of proline and soluble sugar, decreased the accumulation of reactive oxygen species (ROS), and increased the expressions of stress-related genes [43]. Compared with the wild type, the overexpression of the *CaNAC46* gene identified in pepper improved the drought and salt tolerance of transgenic *A. thaliana*, enhanced both root elongation and lateral root development under long-term drought and high salt stresses, reduced the accumulation of ROS, and promoted the expression of SOD, peroxidase (POD), and pyrroline-5-carboxylate synthase (P5CS) [44].

In this study, the soybean transcription factor *GmNAC3* gene was cloned from the drought tolerant soybean variety “Jiyu47”, with the molecular characteristics and the drought tolerant function of GmNAC3 protein further explored by bioinformatics analysis. The expression patterns of the *GmNAC3* gene and a group of five drought-related physiological indices, i.e., the enzymatic activities of SOD, POD, and catalase (CAT) and the contents of proline and malondialdehyde (MDA), were detected in both the transgenic soybean hairy roots and *A. thaliana* plants with the overexpression of *GmNAC3* under drought stress. The results showed that the overexpression of the *GmNAC3* gene significantly improved the drought resistance in soybean. Our study provided novel experimental evidence and candidate genes for further molecular breeding of stress-resistant soybean varieties.

## 2. Results

### 2.1. Gene Cloning of GmNAC3

The total RNA of the roots of soybean seedlings was extracted. The cDNA was synthesized by reverse transcription and used as the template for reverse transcription PCR (RT-PCR) amplification. The PCR product was detected by 1% agarose gel electrophoresis with a clear band between 1000 and 2000 bp (data not shown). The band was recovered and ligated with the cloning vector pMD18-T and transformed into *Escherichia coli* DH5α. The single colonies were randomly picked to extract plasmids for plasmid PCR verification. The results of sequencing were consistent with the Glyma.04G213300 derived from the Phytozome database, indicating that the *GmNAC3* gene of 1452 bp in length was successfully cloned (Table 1). These results indicated the successful construction of the *GmNAC3* recombinant plasmid.

### 2.2. Molecular Properties of Proteins Encoded by GmNAC3 Gene

**Figure 1:**
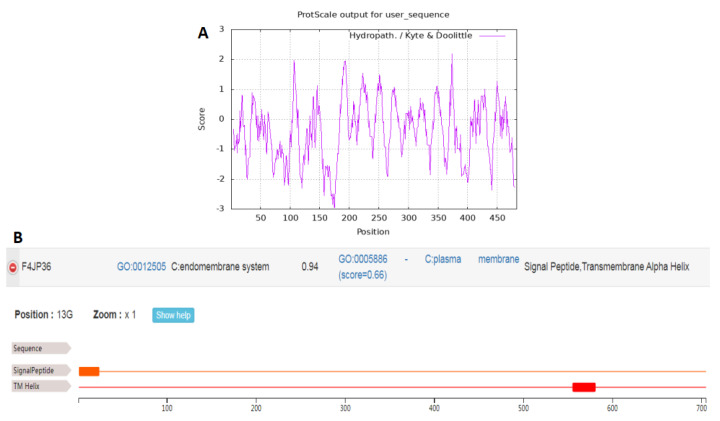
Hydrophobicity (**A**) and subcellular localization (**B**) of GmNAC3 protein based on ProtScale and ProtComp, respectively.

The molecular properties of the GmNAC3 protein were predicted by the ProtParam database. The results showed that the *GmNAC3* gene encoded a protein of a total of 483 amino acids with a molecular weight of 53.6 kDa and the theoretical isoelectric point of 4.81. The instability index of 47.29 indicated that the GmNAC3 protein was unstable, while the average hydrophobic index of –0.413 suggested its high hydrophilicity. The hydrophobicity analysis based on ProtScale (Figure 1A) further supported the hydrophilic property of the GmNAC3 protein with no transmembrane region of GmNAC3 protein identified based on the TMHMM. Based on SignalP, no signal peptide was detected in GmNAC3 protein, indicating that GmNAC3 was not a type of secretory protein. The GmNAC3 protein was located in the nucleus, based on the subcellular localization analysis using ProtComp (Figure 1B).

**Figure 2:**
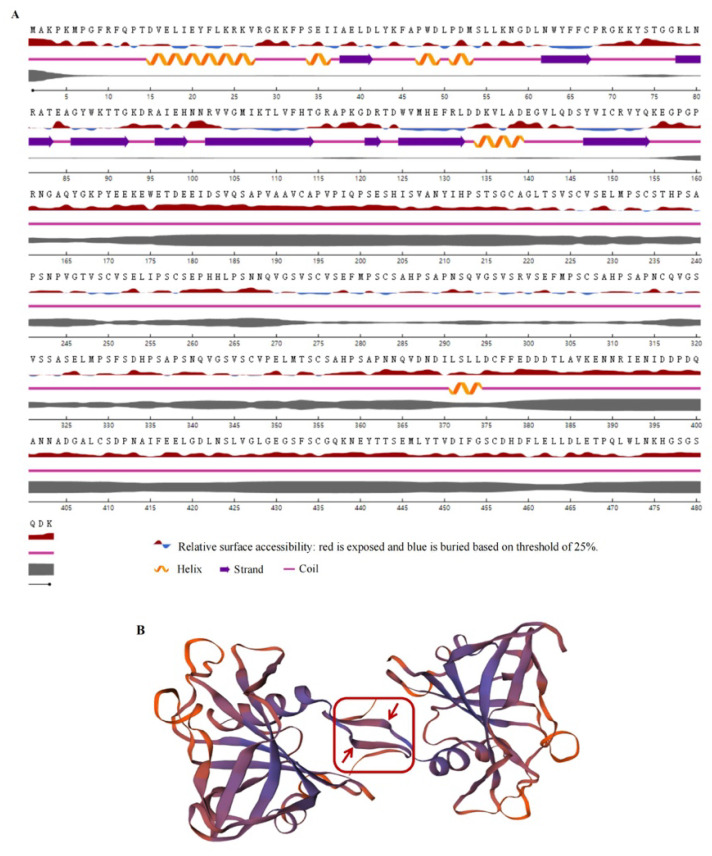
Secondary and tertiary structures of GmNAC3 protein. (**A**) Secondary structure of GmNAC3 protein based on NetSurfP-3.0. Thickness of line represents the probability of disordered residue. (**B**) Tertiary structure of GmNAC3 protein dimer based on and SWISS-MODLE. The red box circumscribes the antiparallel β-sheets forming dimerization interface indicated by red arrows.

The secondary and tertiary structures of the GmNAC3 protein were predicted by NetSurfP-3.0 and SWISS-MODLE, respectively (Figure 2). The results of the secondary structure of GmNAC3 protein showed that α-helix, extended chain, β-turn, and random coil accounted for 18.84%, 11.39%, 5.59%, and 64.18%, respectively, of the total amino acids. The tertiary structure of the GmNAC3 protein was mainly composed of irregular coils connected by several α-helices, which was consistent with the secondary structure of the GmNAC3 protein. The conserved domain of the GmNAC3 protein was identified by SMART as the Pfam domain of the NAM subfamily, covering the amino acids at positions 6 to 132.

**Figure 3:**
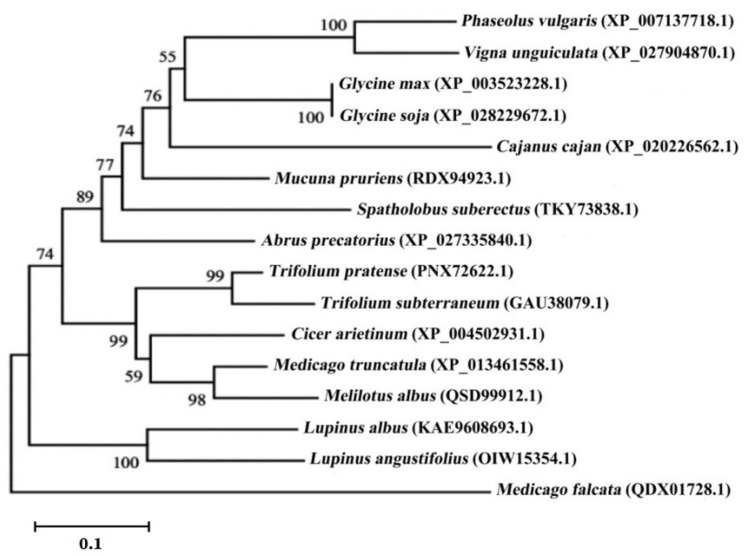
The phylogenetic tree based on the amino acid sequences of a total of 15 NAC proteins and GmNAC3 protein using the neighbor-joining method of MEGA11. GenBank accessions are given in the parentheses after the species names. Bootstrap values based on 1000 replicates are given next to the branches.

**Figure 4:**
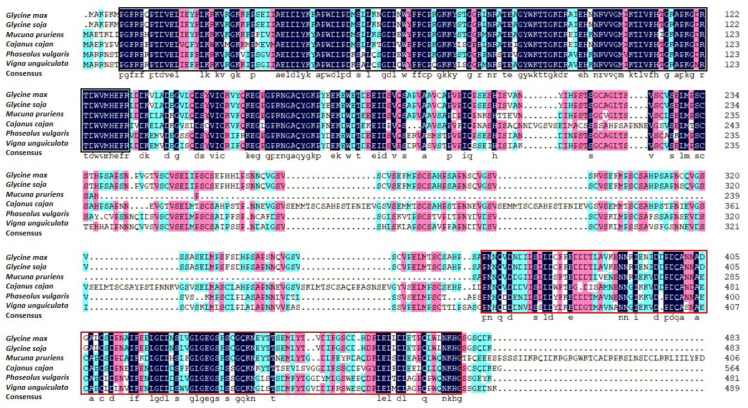
Comparative sequence analysis of GmNAC3 protein and four NAC proteins in five leguminous species based on DNAMAN. The NAM and the activation domains are indicated by the black and red boxes, respectively.

The results of a basic local alignment search tool (BLAST) search based on the GmNAC3 protein sequence identified a total of 15 protein sequences, with the highest homologous similarities to the GmNAC3 protein in the NCBI database (https://blast.ncbi.nlm.nih.gov/; accessed on 12 July 2022). The phylogenetic tree was constructed based on these protein sequences using the neighbor-joining method (Figure 3). The results showed that the GmNAC3 protein was closely related to the wild soybean (Glycine soja) NAC protein sequence, forming a clade with a high amino acid similarity of 99.59%. Both of the two species of Glycine were closely related to the clade composed of *Phaseolus vulgaris* and *Vigna unguiculata*. The multiple sequence comparison of the GmNAC3 protein sequence and the NAC sequences of four species of legumes based on DNAMAN (Figure 4) revealed the relatively conserved region between the amino acids, ranging from positions 8 to 132. These results were consistent with those derived from the SMART prediction of the conserved domains of the GmNAC3 protein.

### 2.3. Transcriptional Activation Activity of GmNAC3 Protein

**Figure 5:**
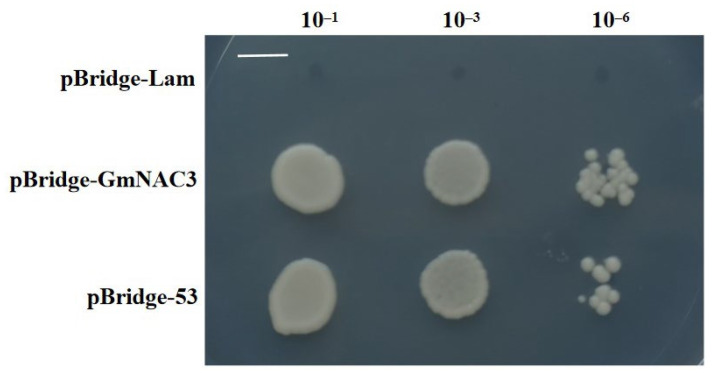
Transcriptional activation activity of GmNAC3 based on *Saccharomyces cerevisiae* strain Y2HGold at three different concentrations (10^–1^, 10^–3^, and 10^–6^). pBridge–Lam vector: negative control not grown on the SD-Trp-His selective medium; pBridge–53: positive control; pBridge–GmNAC3: experimental group. Bar = 1 cm.

The PCR amplification product of the target gene *GmNAC3* was ligated with the pBridge yeast expression vector and transformed into *E. coli* DH5α. The single bacterial colonies were picked and cultured to extract the plasmids. After the target DNA fragment was verified by plasmid PCR electrophoresis, the bacterial liquid was used for sequencing. The sequencing results were consistent with the *GmNAC3* gene sequence, indicating that the pBridge–GmNAC3 vector was successfully constructed. After the recombinant plasmid pBridge–GmNAC3 was transformed into yeast-receptive cells of the *Saccharomyces cerevisiae* strain Y2HGold, the bacterial solution was plated on SD/-Trp selective medium. A total of 3–5 single colonies were picked and mixed, diluted, and plated on the SD/-Trp-His selective medium (Figure 5). The results showed that the yeast cells transformed with negative control pBridge-Lam could not grow on SD/-Trp-His selective medium, while both the yeast transformed with positive control pBridge-53, and the bacterial strain transformed with pBridge-GmNAC3 plasmid, grew normally on the SD/-Trp-His selective medium, indicating the transcriptional activation activity of the GmNAC3 protein.

### 2.4. Subcellular Localization of GmNAC3 Protein

**Figure 6:**
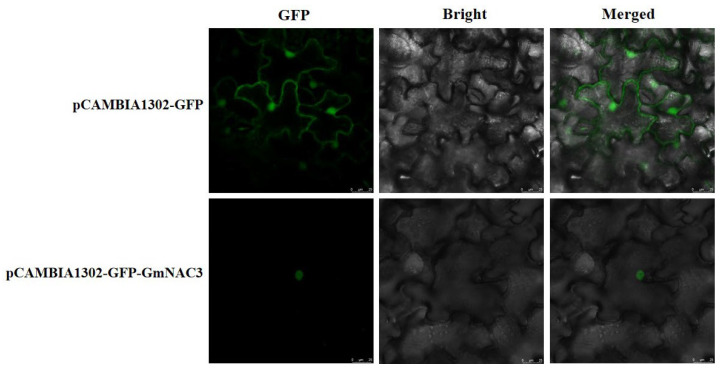
Subcellular localization of GmNAC3 based on the transgenic *Nicotiana benthamiana* showing the transient expression of GmNAC3 fusion protein and green fluorescent protein (GFP) observed using the laser confocal microscopy. GFP: GFP fluorescence; Bright: bright field; Merged: the superposition of both bright and GFP fluorescence fields. pCAMBIA1302–GFP: control; pCAMBIA1302–GFP–GmNAC3: experimental group.

The PCR amplification product of the target gene *GmNAC3* was ligated with the pCAMBIA1302–GFP vector and transformed into *E. coli* DH5α. The single bacterial colonies were cultured to extract the plasmids. After the target DNA fragment was verified by plasmid PCR electrophoresis, the bacterial liquid was used for sequencing. The sequencing results were consistent with the *GmNAC3* gene sequence, indicating the successful construction of the pCAMBIA1302–GFP–GmNAC3 vector, which was first transformed into *Agrobacterium tumefaciens* GV3101 and then transformed into *Nicotiana benthamiana*. The position of the fusion protein was observed using the fluorescence confocal microscope (Figure 6). The results revealed fluorescence in the nuclei and nuclear membranes of *N. benthamiana* in the control group, while the green fluorescent protein (GFP) was only observed in the nucleus of transgenic *N. benthamiana* plants with *GmNAC3*. These results showed that the GmNAC3 protein was localized in the nucleus, which was consistent with results derived from the subcellular localization analysis based on ProtComp (Figure 1B).

### 2.5. Expression Pattern of GmNAC3 Gene

**Figure 7:**
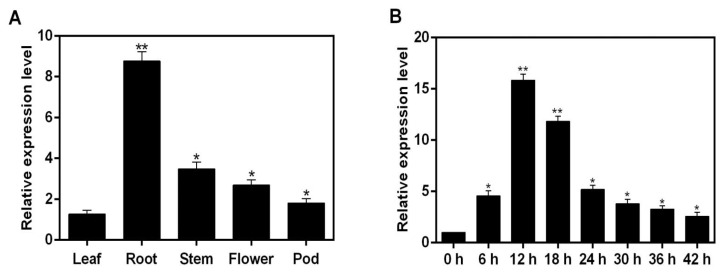
Expressions of *GmNAC3* gene in different organs of soybean relative to that of leaf (**A**) and in roots of soybean seedlings treated with 20% PEG6000 for 42 h relative to 0 h (**B**) based on the real-time fluorescence quantitative real-time PCR. Symbols “*” and “**” indicate significant differences at *p* values of 0.05 and 0.01, respectively.

Using the cDNA based on soybean roots, stems, leaves, flowers, and pods as templates, the expressions of the *GmNAC3* gene were quantitatively evaluated by real-time fluorescence quantitative PCR. The results showed that the lowest expression level of *GmNAC3* was revealed in leaves, while the highest expression was detected in roots, which was 7.5 times higher than that in leaves (Figure 7A). In order to further explore the expression of *GmNAC3* gene in soybean roots under drought conditions, the relative expressions of *GmNAC3* gene were determined in roots of the soybean seedlings treated with 20% PEG6000 (Figure 7B). The results showed that the expression of the *GmNAC3* gene was increased to the highest level in 12 h after the treatment, which was 16 times higher than that of the control. From 12 to 42 h after the treatment, the expressions of the *GmNAC3* gene were decreased to the lowest level at 42 h, which was ~3 times higher than that of the control.

### 2.6. Generation of Transgenic Soybean Hairy Roots and Arabidopsis thaliana with GmNAC3

The full and round seeds of drought tolerant soybean variety “Jiyu47” were sown with the intervals of 1–2 cm in the mixture of nutrient soil:vermiculite (1:1; *v*/*v*), kept in shade for 6–8 d. When the seeds germinated before the cotyledons were fully expanded, the *Agrobacterium rhizogenes* K599 transformed by recombinant plasmid pCAMBIA3301–GFP–GmNAC3 was injected with a needle into the middle portion of hypocotyl of the soybean cotyledonary node. The seedlings were transferred to Hoagland nutrient solution for water culture with the cotyledons removed when the hairy roots were observed. In 15–20 days, when the hairy roots elongated to 3–5 cm, the main roots were removed and the culture was restored. A group of 8 hairy roots at similar developmental stages were selected to extract genomic DNA to perform the PCR analysis, based on the primers of 35S promoter (776 bp) and *bar* gene (488 bp), which were contained in the pCAMBIA3301–GFP vector, to detect the positive hairy roots (data not shown). The target DNA fragments were observed in all 8 hairy roots, suggesting that these hairy roots of the *GmNAC3* transgenic soybean seedlings were all positive.

The recombinant plasmid pCAMBIA3301–GFP–GmNAC3 was then transformed into *Agrobacterium tumefaciens* AGL0. The seeds of *Arabidopsis thaliana* were transformed by *Agrobacterium tumefaciens* dipping method and sprayed with the Basta herbicide after germination. The transgenic *A. thaliana* with *GmNAC3* gene was obtained and grown into T3 generation. The genomic DNA with transgenic *GmNAC3* gene was extracted to perform the PCR analysis based on the 35S promoter and *bar* gene to detect the positive plant materials (data not shown).

**Figure 8:**
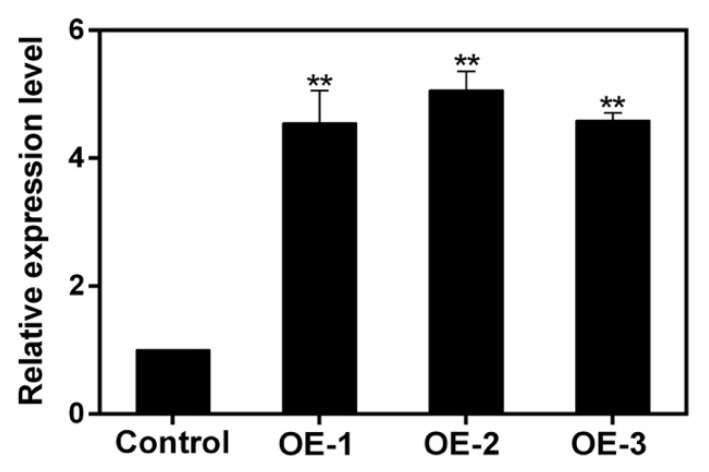
Overexpression of *GmNAC3* in three lines of transgenic soybean hairy roots (OE-1, OE-2, and OE-3) relative to control based on quantitative real time PCR (qRT-PCR) analysis. Control: soybean hairy roots transfected with *Agrobacterium rhizogenes* K599. Symbols “**” indicate significant differences at *p* value of 0.01.

Three lines of soybean hairy roots with overexpression of *GmNAC3* gene were selected for quantitative real-time PCR (qRT-PCR) analysis (Figure 8). The expression level of the *GmNAC3* gene was over 4 times higher than that of the *Agrobacterium rhizogenes* K599-transfected hairy roots, indicating the overexpression of *GmNAC3* gene in these soybean hairy roots.

### 2.7. Stress Resistance of Soybean Hairy Roots and Expression of Drought-Related Genes in Transgenic Soybean with GmNAC3

**Figure 9:**
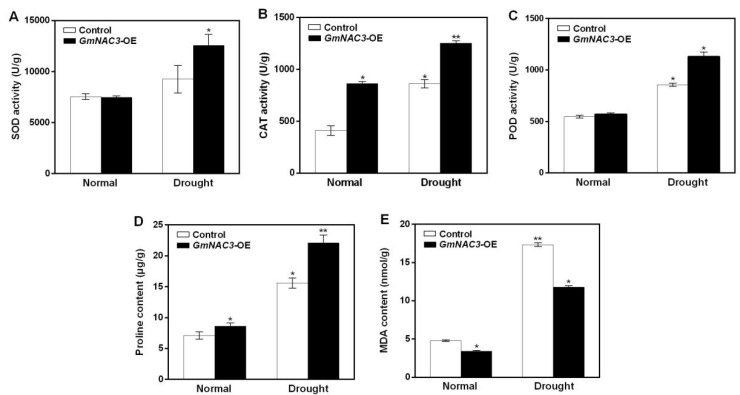
Physiological indices of soybean hairy roots under normal growth condition and drought stress for 12 h, including the enzymatic functions of superoxide dismutase (SOD) activity (**A**), catalase (CAT) activity (**B**), peroxidase (POD) activity (**C**), and the proline content (**D**) and malondialdehyde (MDA) content (**E**). Symbols “*” and “**” indicate significant differences at *p* values of 0.05 and 0.01, respectively.

A group of five physiological indices, i.e., enzymatic activities of SOD, POD, and CAT and the contents of proline and MDA, related to drought resistance were determined in soybean hairy roots treated with 20% PEG6000 for 12 h to evaluate the functions of *GmNAC3* in soybean response to drought stress (Figure 9). The results showed that under normal (non-drought) growth conditions, the CAT activity in the hairy roots of transgenic soybean was significantly higher than that of the control, the content of MDA was lower than that of the control, while the activities of SOD and POD and the proline content in the hairy roots of transgenic soybean were not significantly different from those of the control. In 12 h after the drought treatment, the activities of SOD and POD were increased to 1.3 and 1.4 times higher than that of the control, respectively, the CAT activities and proline content were significantly increased to 1.45 and 1.4 times higher than that of the control, whereas the MDA content was significantly decreased to 68% lower than that of the control. These results indicated that the *GmNAC3* gene enhanced the activities of antioxidant enzymes in soybean under drought stress.

**Figure 10:**
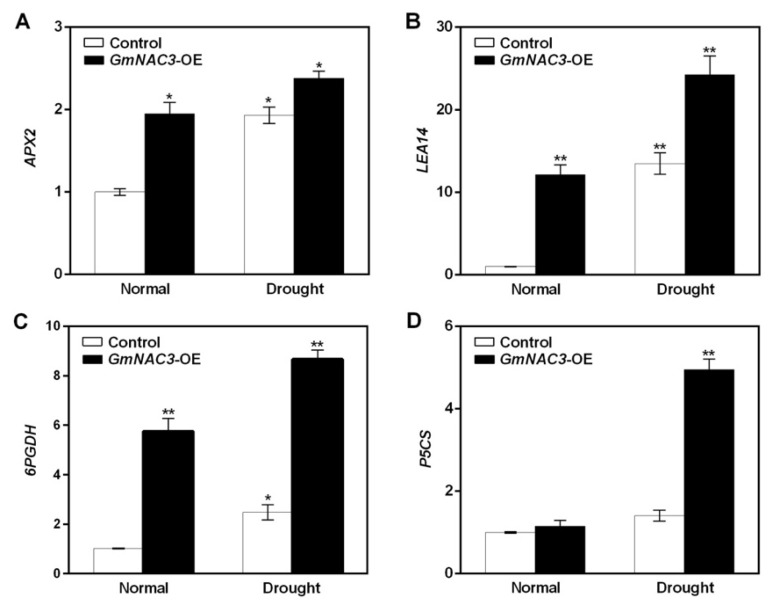
Relative expression of four drought response-related genes, i.e., *APX2* (**A**), *LEA14* (**B**), *6PGDH* (**C**), and *P5CS* (**D**), in transgenic soybean plants with the overexpression of *GmNAC3* gene under drought stress relative to the control under normal growth condition (non-drought) based on quantitative real-time PCR analysis. Symbols “*” and “**” indicate significant differences at *p* values of 0.05 and 0.01, respectively.

The transcriptional levels of a group of four drought-stress-related genes, including *APX2*, *LEA14*, *6PGDH*, and *P5CS*, were further detected to explore the molecular response to drought stress of the *GmNAC3* gene under drought treatment based on qRT-PCR analysis (Figure 10). The results showed that under normal growth condition, the transcriptional levels of *APX2*, *LEA14*, and *6PGDH* in transgenic soybean plants with overexpression of the *GmNAC3* gene were significantly or very significantly higher than those with empty vectors (i.e., control), while the transcriptional level of P5CS was significantly increased. Under drought stress, compared with soybean seedlings with an empty vector, the expression levels of *APX2*, *LEA14*, *6PGDH*, and *P5CS* were significantly up-regulated in the transgenic soybean plants with overexpression of the *GmNAC3* gene and were increased to 1.3, 1.7, 3.5, and 3.3 times higher that of the control, respectively.

### 2.8. Phenotypic Variations of Transgenic Arabidopsis thaliana with GmNAC3 under Drought Stress

**Figure 11:**
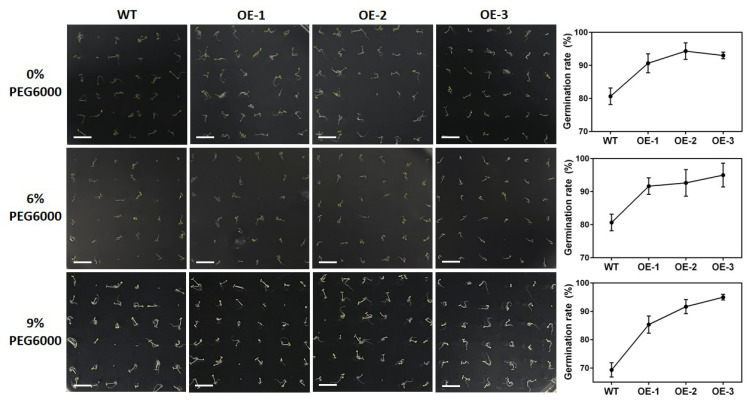
Germination rates of three lines of transgenic *Arabidopsis thaliana* with overexpression of *GmNAC3* (OE-1, OE-2, and OE-3) treated with PEG6000 of three concentrations (i.e., 0%, 6%, and 9%), respectively. Bar = 1 cm.

**Figure 12:**
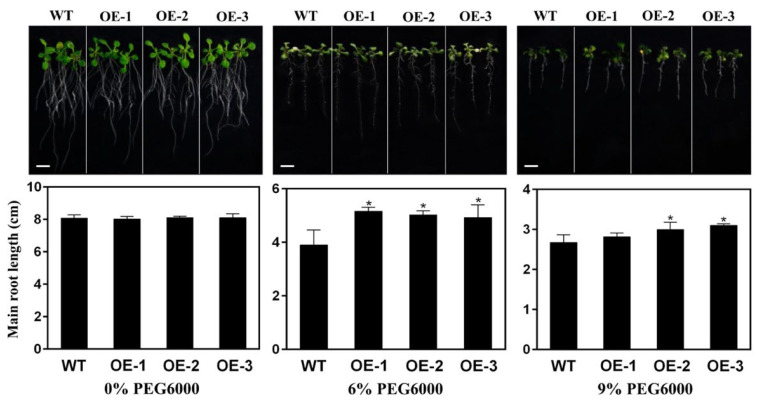
Root growth of three lines of transgenic *Arabidopsis thaliana* with overexpression of *GmNAC3* (OE-1, OE-2, and OE-3) treated with PEG6000 of 0%, 6%, and 9%, respectively. The plants shown in the top panel correspond to the bar graphs presented in the bottom panel, respectively. Symbol “*” indicates significant differences at *p* values of 0.05 and 0.01, respectively. Bar = 1 cm.

The seeds of both wild type and transgenic *A. thaliana* were sown on Murashige and Skoog (MS) medium with PEG6000 of 0%, 6%, and 9%, respectively (Figure 11). The results showed that the germination rates of transgenic *A. thaliana* seeds on the medium with PEG6000 of three concentrations (i.e., 0%, 6%, and 9%) were 92%, 93%, and 91%, respectively, while the germination rates of wild type *A. thaliana* were 81%, 81%, and 69% on the medium with PEG6000 of 0%, 6%, and 9%, respectively. The average root lengths of transgenic *A. thaliana* were 8.1 cm, 5.0 cm, and 3.0 cm, and the average root lengths of wild type *A. thaliana* were 8.1 cm, 3.9 cm, and 2.7 cm, on the medium with PEG6000 of 0%, 6%, and 9%, respectively (Figure 12). Compared with the wild type, the germination rate of transgenic *A. thaliana* seeds was increased under drought stress with varied concentrations of PEG6000. Although the root elongation of transgenic *A. thaliana* was inhibited by drought stress, the roots of transgenic *A. thaliana* were longer than those of wild type, while the taproot of *A. thaliana* with overexpression of *GmNAC3* gene grew more vigorously under drought stress than that under normal growth conditions. These results suggested that the overexpression of *GmNAC3* gene promoted the seed germination and root growth of *A. thaliana* and improved the drought resistance in *A. thaliana*.

**Figure 13:**
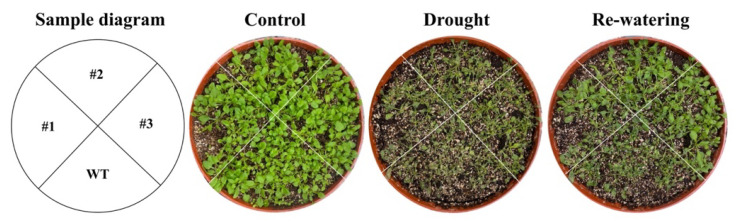
Results of drought resistance experiments of wild type (WT) and three lines of transgenic *Arabidopsis thaliana* plants with overexpression of *GmNAC3* (#1, #2, and #3) under normal growth condition (control), drought stress (i.e., treatment of 12% PEG6000), and recovery of drought stress by re-watering.

No significant phenotypic variations were observed in the growth of wild type and transgenic *A. thaliana* under the normal growth conditions (Figure 13). Under the drought stress (i.e., treatment of 12% PEG6000), the wilting degree of wild type *A. thaliana* was significantly higher than that of the transgenic plants, while the recovery ability of the transgenic plants was stronger than that of wild type after re-watering (Figure 13).

### 2.9. Physiological Indices of Transgenic Arabidopsis thaliana with GmNAC3 under Drought Stress

The physiological indices related to stress resistance were further investigated in transgenic and wild type *A. thaliana* plants (Table 2). The results showed that under normal growth conditions, the physiological indices of transgenic *A. thaliana* were all higher than those of wild type plants. Under the drought treatment, the enzymatic activities of SOD, POD, and CAT and the proline content in transgenic *A. thaliana* were higher than those in the wild type plants, whereas the content of MDA in transgenic *A. thaliana* was lower than that in the wild type plants. Specifically, the SOD activity and proline content of transgenic *A. thaliana* were very significantly higher than those of wild type plants, and the activities of CAT and POD in transgenic were significantly higher than those of wild type plants, which were 8.2, 2.5, 1.4 and 2.1 times higher than those in the wild type plants, respectively. The MDA content in transgenic *A. thaliana* was significantly decreased to ~50% of that in the wild type plants.

## 3. Discussion

It is well-known that as the indispensable regulatory proteins in plants, the transcription factors, play important roles in regulating gene expression at the transcriptional level in response to drought stress. NAC is one of the largest families of plant-specific transcription factors, which plays key roles in the molecular mechanism regulating plant response to drought stress [9] with the specificity domain of NAC bound specifically to the CaMV35S promoter, while the C-terminal of NAC is a transcriptional regulatory region that controls the transcriptional activation [27]. Studies showed that three soybean genes (i.e., *GmNAC085*, *GmNAC092*, and *GmNAC109*) were induced to express in soybean roots under drought stress and to improve the drought tolerance of soybean [26]. In our study, the soybean *GmNAC3* gene was cloned with the molecular properties of both *GmNAC3* and GmNAC3 further characterized. The open reading frame of the *GmNAC3* gene was 1452 bp in length, encoding the GmNAC3 protein of a total of 483 amino acids with the molecular weight of 53.6 kDa and the theoretical isoelectric point of 4.81. The GmNAC3 was an unstable hydrophilic protein without signal peptide, showing high homologous similarity with wild soybean (i.e., 99.59% similarity at amino acid level) and shared structural characteristics of NAC transcription factors, including the presence of NAM domain [45]. These results suggested the candidacy of GmNAC3 as a transcription factor involved in the molecular response to drought stress in soybean.

To further explore the molecular functions of the *GmNAC3* gene in the regulation of the molecular response to drought stress in soybean, the transcriptional activation activity of GmNAC3 was revealed using subcellular localization experiments based on transgenic plants of *N. benthamiana* with the overexpression of *GmNAC3*. The results of the subcellular localization experiment of *N. benthamiana* and the yeast activation experiment showed that the GmNAC3 protein was located in the nucleus, with the transcriptional activation function in response to drought stress. Similarly, studies have shown that several NAC transcriptional factors were located in the nucleus with transcriptional activation activities identified in various crop plants, e.g., ONAC066 [43], GmNAC109 [46], GmNAC065, GmNAC085, and GmNAC177 [47], OsNAC3 [48], and GmNAC5 and GmNAC6 [45].

A large number of studies have shown that a variety of transcription factors are specifically expressed in plant roots to improve the root growth and to enhance the crop drought resistance. For example, the transcription factor WRKY33 was highly expressed in *A. thaliana* roots to mediate the phosphate deficiency-induced remodeling of root architecture by modulating iron homeostasis [49], while the response pattern of auxin was changed in the roots of *A. thaliana* seedlings with the overexpression of ATHB2 [50]. Furthermore, TaRNAC1 was highly expressed in wheat roots to enhance root length, biomass, and drought tolerance in wheat [51]. These studies were consistent with the results revealed in our study, showing that most transcription factors played important roles in root development. In our study, the highest level of expression of the *GmNAC3* gene was detected in soybean roots, which was 7.5 times higher than that in leaves. Furthermore, our results showed that the expression of *GmNAC3* gene in soybean roots treated with 20% PEG6000 to simulate drought stress was initially increased to the highest level in 12 h after the treatment, which was 16 times higher than that of the control. These results were consistent with those reported previously. For example, under the PEG6000 treatment, the expression of the *JrMYB44* gene in walnut was initially increased and then decreased over time [52]. Similarly, under drought stress (i.e., treatment of PEG6000), the expression of the *OsbZIP62* gene in rice was initially increased to the highest level in 12 h after the treatment and then decreased [53]. Further investigations are needed to explore the regulatory functions of GmNAC3 in soybean under drought stress.

As one of the components in the normal metabolism of the biological systems, the production of ROS is enhanced by adverse environmental factors, such as drought stress, to a level that is harmful to plants [54,55]. To alleviate the detrimental effects caused by ROS, antioxidant enzymes play an important role in decreasing the levels of ROS and oxidative stress [56,57]. Furthermore, a large number of studies have revealed the cytotoxic effect of MDA, i.e., inhibiting gene expression and promoting cell death. For example, under drought stress, a large amount of MDA is produced in plants with deleterious effect [58]. In addition to the antioxidant enzymes, the content of free proline is also increased to improve the stress resistance of plants under drought, salt, or cold stresses [59,60]. Moreover, studies showed that with the overexpression of *BpMYB123* in *Betula platyphylla*, the activities of both SOD and POD were increased, while the content of MDA was decreased under drought stress [61]. Similarly, the content of MDA was significantly decreased in transgenic *A. thaliana* with overexpression of *VvNAC17* under drought treatment, while the activities of SOD, POD, and CAT and the content of proline were significantly increased [62]. These results were consistent with the findings revealed in our study, showing that the activities of SOD, POD, and CAT and the content of proline in the transgenic *A. thaliana* plants and soybean hairy roots with the overexpression of *GmNAC*3 were higher than those in the control group, while the MDA content was lower than that in the control group. These results suggested that the *GmNAC3* gene could increase the activities of antioxidant enzymes to remove the accumulation of ROS, reduce the content of MDA, and increase the content of proline to enhance the osmotic regulation, ultimately increasing the drought tolerance of transgenic plants. Future studies are necessary to identify the functions of these antioxidant enzymes and proline in the plant response to drought stress in soybean.

It is well-known that multiple drought resistance mechanisms are triggered under drought stress, such as enhanced production of osmoregulatory substances or improved antioxidant capacity, with numerous genes involved. For example, studies showed that the gene knockout of *P5CS* in *Arabidopsis* caused reduced levels of proline synthesis and increased accumulation of reactive oxygen [63], while the overexpression of the mothbean *P5CS* gene increased the proline content in transgenic tobacco plants, enhanced the permeability, and improved the plants’ drought resistance [64]. Furthermore, LEA proteins prevent water loss in cells during drought stress due to their high hydration capacity. For example, studies showed that the expressions of both *P5CS* and LEA were increased in rice by the overexpression of the *OsWRKY50* gene under salt stress [65]. Moreover, it was reported that the expression of *APX2* was up-regulated and the accumulation of ROS was reduced in the transgenic *Arabidopsis* with the overexpression of the *csWRKY33* gene under drought stress [66]. Additionally, as a key enzyme in the pentose phosphate pathway, 6PGDH has been revealed with increased activity to enhance the stress resistance of plants. For example, *Os6PGDH* was up-regulated in rice seedlings under salt stress [67]. These results were consistent with the findings revealed in our study, showing that the overexpression of *GmNAC3* caused the up-regulated expressions of *APX2*, *P5CS*, *LEA14*, and *6PGDH* in soybean plants, suggesting that *GmNAC3* was involved in the regulation of the osmoregulatory function in plants under drought stress by enhancing the expressions of these stress resistance-related genes. Future studies are needed to identify the explicit functions of these genes in the molecular response to drought stress in soybean.

Phenotypic variations could be directly observed to show the changes in plant growth and development before and after the treatment of environmental stress. For example, both the root length and germination rate were increased in transgenic rice with *OsNAC3* [68]. Similarly, the germination rate, root length, and improvement of growth and development were observed in transgenic maize with *ZmbZIP4* under drought and salt stresses [69]. Furthermore, studies showed that the length of hairy roots of transgenic soybean with *GmbZIP2* was significantly longer than that of the control group under drought and salt stresses [70]. Moreover, both the germination rate and root length of *A. thaliana* with overexpression of *GmWRKY16* under osmotic stress were increased [71]. Studies have shown that the overexpression of peroxisome-localized *GmABCA7* of soybean promoted seed germination via the β-oxidation of fatty acids in *Arabidopsis thaliana* [72], while the transcription factor NAC103 improved seed germination by regulating several abscisic acid (ABA)-responsive downstream genes in *Arabidopsis* [73]. Moreover, the seed germination rate of transgenic *Arabidopsis thaliana* with overexpression of *GmG6PD7* under NaCl treatment was enhanced, with the up-regulation and down-regulation observed in ABA degradation genes and ABA synthesis/responsive genes, respectively, leading to reduced ABA content [74]. Additionally, the overexpression of *OoNAC72* of *Oxytropis ochrocephala* caused ABA hypersensitivity and enhanced drought tolerance during seed germination in *Arabidopsis* as well as the expression of stress-responsive genes, e.g., *RD29A*, *RD29B*, *RD26*, *LEA14*, *ANACOR19*, *ZAT10*, *PP2CA*, and *NCED3* [75]. Future studies are necessary to further explore the explicit functions of these genes in the seed germination of transgenic crop plants with overexpression of *GmNAC3*. These results were consistent with the findings revealed in our study, showing increased germination rate and root length, enhanced drought tolerance, and improved recovery ability after rehydration in transgenic *A. thaliana*. These results suggested that the *GmNAC3* gene was involved in the molecular response to drought stress by regulating the expressions of drought-related genes, further enhancing the growth and development in plants under drought stress. Future studies are necessary to confirm the findings revealed in our study and to further explore the specific functions of *GmNAC3* in the molecular response to drought stress in soybean.

Our study showed that the overexpression of the *GmNAC3* gene enhanced the drought resistance in both soybean roots and *A.* *thaliana*, and these results were consistent with those derived from the quantitative trait locus (QTL) and the genome-wide association study (GWAS) analyses of NAC transcription factors involved in crop drought resistance. For example, a recent study showed that a total of nine candidate genes involved in drought tolerance were identified in a QTL of a soybean recombinant inbred line and were further annotated as genes encoding the NAC transport factor and GATA transport factor proteins [76]. Furthermore, QTLs and candidate genes involved in physiological traits and drought tolerance were identified in cotton, e.g., a group of microRNAs were closely associated with *NAC* and *MYB* genes, playing a profound role in enhancing drought tolerance in cotton [77]. Moreover, the genome-wide QTL analysis of peanut revealed a total of 19 QTLs associated with drought tolerance, with genes encoding transcription factors such as MADS-box, basic helix–loop–helix (bHLH), NAM, and NAC, involved in peanut growth, development of seed and pod, and photosynthesis under drought conditions [78]. Additionally, a previous GWAS study revealed the wheat *NAC* gene (i.e., *TaNAC071-A*) was closely associated with drought tolerance, i.e., the knockdown of *TaNAC071-A* attenuated drought tolerance of wheat, whereas its overexpression significantly enhanced drought tolerance through improved water-use efficiency and increased expression of stress-responsive genes [79]. Another genome-wide expression analysis in rice revealed novel candidate genes involved in water stress adaptation, including members in the families of NAC, AP2/ERF, WRKY, and MYB playing important roles in drought adaptation [80]. These results indicated that the identification of these QTLs and genes involved in drought tolerance would significantly facilitate the molecular breeding of drought tolerant soybean plants.

## 4. Materials and Methods

### 4.1. Materials and Reagents

Seeds of the drought tolerant soybean variety “Jiyu47” were sown in a mixture of nutritious soil and vermiculite (1:1, *v*/*v*). The young roots of soybean plants with trifoliate leaflets observed were collected for cloning the *GmNAC3* gene. The 7-day-old soybean seedlings were transferred to Hoagland nutrient solution with 20% PEG6000, which was used to simulate drought stress. The roots were sampled every 6 h to detect the *GmNAC3* gene expression under drought stress. Soybean plants with mature cotyledons of 7 days old were infected with *Agrobacterium rhizogenes* K599 and transferred to Hoagland nutrient solution. The cotyledons were removed when hairy roots were observed. The plants were continuously cultured for 15–20 days, then the main roots were collected and moved to nutrient solution with 20% PEG6000 for 12 h to be used for drought-related experiments. The growth chamber was maintained under a photoperiod cycle of 12 h light and 12 h dark at 22 °C with a relative humidity of 50% and a light intensity of 310 μmol m^–2^ s^–1^. Soybean seeds were sown and plants were grown in the experimental site of Jilin Agricultural University with roots, stems, leaves, flowers, and pods during the podding stage collected for the *GmNAC3* gene expression.

After vernalization, seeds of wild type *A. thaliana* ecotype Columbia (Col-0) were sown in the mixture of nutritious soil and vermiculite (1:3, *v*/*v*). The *GmNAC3* gene transformation was completed by floral dipping method at the flowering stage of the soybean plants. The *GmNAC3* transgenic *A. thaliana* plants in T3 generation were grown on Murashige and Skoog (MS) medium with PEG6000 of three concentrations (i.e., 0%, 6%, and 9%), respectively, to evaluate the germination rate and root length of *A. thaliana* under drought conditions. The *A. thaliana* plants at seedling stage were treated with 12% PEG6000 for 24 h and then rehydrated to observe their phenotypic variations. The *GmNAC3* transgenic *A. thaliana* plants were grown on MS medium of 9% PEG6000 and sampled in 20 d for drought-related experiments. The growth chamber was maintained with a photoperiod cycle of 16 h light and 8 h dark at 22 °C with a relative humidity of 60% and a light intensity of 310 μmol m^–2^ s^–1^.

The seeds of *Nicotiana benthamiana* were sown in a mixture of nutritious soil and vermiculite (3:1, *v*/*v*). In 4 weeks, the plants of *N. benthamiana* were used for subcellular localization experiments. The growth chamber was maintained with a photoperiod cycle of 16 h light and 8 h dark at 22 °C with a relative humidity of 50% and a light intensity of 360 μmol m^–2^ s^–1^.

Reagents, vectors, and cell culture materials used in this study included: *Escherichia coli* competent cell DH5α (Tiangen Company, Beijing, China), yeast competent cell of *Saccharomyces cerevisiae* strain Y2HGold (Coolaber Company, Beijing, China), *Agrobacterium tumefaciens* competent cell GV3101 (Coolaber Company, Beijing, China); restriction endonucleases (i.e., BamHI, HindIII, BglII, and SpeI), pMD18-T vector, Ex-Taq, RNAiso Plus, RevertAid First Strand cDNA Synthesis Kit, and plasmid extraction and gel recovery kits were purchased from Takara Co., Ltd. (Dalian, China). All other reagents, e.g., PEG6000, glucose, and potassium nitrate (Solarbio Science and Technology Co., Ltd., Beijing, China), were of analytical grade. The primers and DNA sequencing were synthesized and performed, respectively, by Shanghai Biotech Bioengineering Co., Ltd. (Shanghai, China). The pBridge vector, pCAMBIA1302–GFP vector, and plant expression vector pCAMBIA3301–GFP as well as the *Agrobacterium tumefaciens* competent cell AGL0 and *A. rhizogenes* competent cell K599 were obtained from the Laboratory of Soil and Plant Molecular Genetics, College of Plant Science, Jilin University, China. The synthetic dropout media (SD/-Trp and SD/-Trp-His selective medium) were purchased from Fun Genome Company (Beijing, China).

### 4.2. Gene Cloning of GmNAC3

The sequence of the soybean *GmNAC3* gene was obtained from the Phytozome (Glyma.0G4213300; https://phytozome-next.jgi.doe.gov/; accessed on 12 June 2022) database with the specific primers designed by Primer Primer5.0 (Table 3). Total RNA was extracted from young soybean roots using the RNAiso Plus by following the manufacturer’s instructions. The cDNA was obtained using the RevertAid First Strand cDNA Synthesis Kit based on the manufacturer’s protocols. The *GmNAC3* gene was amplified by RT-PCR with the amplification procedure as follows: pre-denaturation at 95 °C for 3 min, followed by 35 cycles of denaturation at 95 °C for 30 s, annealing at 60 °C for 30 s, and extension at 72 °C for 75 s, and final extension at 72 °C for 10 min. The PCR amplification products were detected by 1% agarose gel electrophoresis, the target bands were removed using the UV gel cutter, and the DNA target fragments were recovered using the gel recovery kit based on the manufacturer’s instructions. The recovered product was ligated with pMD18-T vector overnight at 16 °C, and the ligated product was transformed using the heat shock method with the *E. coli* DH5α, based on the manufacturer’s instructions. The sample was plated and incubated overnight at 37 °C. The positive single colonies were picked and verified by PCR analysis, and used to obtain the target fragment. The bacterial cultures with positive colonies were used for sequencing.

### 4.3. Properties of GmNAC3 Gene and GmNAC3 Protein

The molecular properties of the *GmNAC3* gene and the GmNAC3 protein were explored by bioinformatics analysis using the online servers, including the physicochemical analysis of protein based on ProtParam (https://web.expasy.org/protparam/; accessed on 12 July 2022), hydrophobicity analysis based on ProtScale (https://web.expasy.org/protscale/; accessed on 12 July 2022), signal peptide analysis based on SignalP (http://www.cbs.dtu.dk/services/SignalP-4.0/; accessed on 12 July 2022), protein secondary structure prediction based on NetSurfP (https://services.healthtech.dtu.dk/service.php?NetSurfP-3.0/; accessed on 12 July 2022), the prediction of protein tertiary structure using the SWISS-MODEL (https://swissmodel.expasy.org/; accessed on 12 July 2022), the prediction of protein structure based on SMART (http://smart.embl-heidelberg.de/; accessed on 12 July 2022); subcellular localization prediction using ProtComp (http://www.softberry.com/; accessed on 12 July 2022), and transmembrane protein prediction analysis based on the tied mixture hidden Markov model (TMHMM; http://www.cbs.dtu.dk/services/TMHMM/; accessed on 12 July 2022). A total of 15 NAC protein sequences of 15 species of crop plants were retrieved by the basic local alignment search tool (BLAST; https://blast.ncbi.nlm.nih.gov/; accessed on 12 July 2022) using the protein sequence of GmNAC3 as the query. The neighbor-joining tree based on these protein sequences was constructed using MEGA11 (https://www.megasoftware.net/; accessed on 12 July 2022). The amino acid sequence alignment of six NAC proteins was performed using DNAMAN (https://www.lynnon.com/; accessed on 12 July 2022).

### 4.4. Transcriptional Activation Activity of GmNAC3

A pair of specific primers with upstream EcoRI restriction site and downstream BamHI restriction site (Table 3) were designed to perform PCR amplification using the pMD-GmNAC3 plasmid as a template. The target bands were collected and ligated with the pBridge vector linearized by EcoRI and BamHI. The single colonies were picked and cultured in LB liquid medium (with kanamycin resistance), verified by liquid PCR, and sequenced. The target plasmid pBridge–GmNAC3, negative control pBridge–Lam, and positive control pBridge–53 were transformed into the yeast strain Y2HGold, respectively, and screened by incubation in the SD/-Trp selective medium at 30 °C for 36 h. The positive single colonies were picked and cultured on the SD/-Trp-His selective medium (with three biological replicates per sample) at 30 °C for 36 h to observe the growth of the transformed yeast colonies.

### 4.5. Subcellular Localization of GmNAC3 Protein

A pair of specific primers with the BglII upstream restriction site and the SpeI downstream restriction site were designed (Table 3) to perform the PCR amplification using the pMD-GmNAC3 plasmid as the template. The target bands were collected and ligated with the pCAMBIA1302–GFP vector linearized by BglII and SpeI. The single colonies were selected and cultured in LB liquid medium, verified by liquid PCR, and sequenced. The sequencing results revealed 100% similarity with the target gene, indicating the successful construction of the pCAMBIA1302–GFP–GmNAC3 transient expression vector. The recombinant plasmid pCAMBIA1302–GFP–GmNAC3 was transformed into *Agrobacterium tumefaciens* GV3101. The bacterial solution was cultured (with the addition of acetosyringone) to reach OD600 = 0.8 and centrifuged at 4 °C and 5000× *g* for 15 min. The supernatant was discarded and the precipitate was washed twice with *Agrobacterium* transformation medium, centrifuged at 5000× *g* and 4 °C for 15 min, and the cells were collected and dissolved with *Agrobacterium* transformation medium, and placed at room temperature for 2 h. A needle was inserted into but not through the back of leaves of *Nicotiana benthamiana* to generate 5–6 holes, which were used to inject the bacterial solution using a 1 mL syringe; the sample was stored in the dark with ventilation for 48 h, and observed with a laser confocal microscopy.

### 4.6. Construction of Plant Overexpression Vector pCAMBIA3301–GFP–GmNAC3 and Genetic Transformation by Agrobacterium

PCR amplification was performed with the pMD-GmNAC3 plasmid as the template and both qGmNAC3-F and qGmNAC3-R as primers (Table 3). The plant expression vector pCAMBIA3301–GFP was linearized with BamHI. The target gene PCR products and vectors were recovered and ligated with the plant expression vector pCAMBIA3301–GFP. The positive clones were selected for bacterial liquid culture, and the plasmids were extracted for PCR verification and sequencing. The recombinant plasmid pCAMBIA3301–GFP–GmNAC3 was transformed into *Agrobacterium rhizogenes* K599 which was transformed into soybean cotyledons to generate soybean hairy roots. The recombinant plasmid pCAMBIA3301–GFP–GmNAC3 was transformed into *A. tumefaciens* AGL0, and the *A. thaliana* with *GmNAC3* gene was obtained by *A. tumefaciens* AGL0 dipping method and cultured to T3 generation.

### 4.7. Expressions of GmNAC3 Gene and Drought Resistance Related Genes

Both Tublin-F and Tublin-R were used as the internal reference gene primers (Table 3) to perform the qRT-PCR using the All-in-One^TM^ qRT-PCR Mix kit, based on the manufacturer’s instructions, to determine the expressions of the *GmNAC3* gene and drought resistance-related genes in various types of samples, including *APX2*, involved in antioxidant activities [81], *LEA14*, involved in cell dehydration and drought response [82], *6PGDH*, related to crop stress tolerance [83], and *P5CS*, involved in the synthesis of osmotic regulator proline [64], in soybean hairy roots. Each experiment was repeated with three biological replicates. The relative gene expression level was calculated according to the measured cycle threshold (Ct value) and the 2^–∆∆^^Ct^ method [84].

### 4.8. Physiological Indices Related to Stress Resistance

The physiological indices, i.e., the activities of SOD, POD, and CAT, as well as the contents of MDA and proline, were determined by kits based on the manufacturer’s protocols (Grace Biotechnology, Suzhou, China). Each experiment was repeated with three biological replicates.

## 5. Conclusions

The gene *GmNAC3* encoding the drought-inducible transcription factor GmNAC3 was cloned from the drought tolerant soybean variety “Jiyu47” with the GmNAC3 protein localized in the nucleus. The *GmNAC3* gene was significantly up-regulated in the transgenic soybean hairy roots under drought stress. The overexpression of the *GmNAC3* gene could up-regulate the expression of stress resistance-related genes and enhance the antioxidant ability in transgenic soybean hairy roots. The germination rate of transgenic *A. thaliana* seeds was improved under drought conditions with promoted rooting and improved recovery ability after rehydration. This study provided strong experimental evidence to support further investigations of the regulatory functions of *GmNAC3* and to enhance the molecular breeding of soybean varieties with increased drought tolerance.

## Figures and Tables

**Table 1:** Chromosomal locations of the *NAC* gene on the reference genomes in a total of 16 species of plants based on the National Center for Biotechnology Information (NCBI) database. Symbol “—” indicates unknown data.

Species (Gene ID or Protein ID)	Chromosome Number	Location
*Phaseolusvulgaris* (18620214)	9	21883166–21887652
*Vigna unguiculata* (114164401)	9	27465669–27470240
*Glycine max* (100781170)	4	48507639–48511991
*Glycine soja* (114410096)	4	48113653–48118040
*Cajanus cajan* (109808119)	11	45241951–45245555
*Cicer arietinum* (101512547)	5	47316071–47320366
*Medicago truncatula* (11423052)	3	43934323–43939382
*Abrus precatorius* (113849838)	—	5562701–5565889
*Spatholobus suberectus* (TKY71273.1)	1	108331–108927
*Lupinus albus* (KAE9608693.1)	8	11700963–11705996
*Lupinus angustifolius* (OIW15354.1)	3	344992–346126
*Trifolium subterraneum* (GAU38079.1)	7	99883–102744
*Trifolium pratense* (PNX72622.1)	2	—
*Mucuna pruriens* (RDX94923.1)	—	32788–36295
*Melilotus albus* (QSD99912.1)	—	—
*Medicago falcata* (QDX01728.1)	—	—

**Table 2:** Variations of physiological indices, i.e., the enzymatic activities of superoxide dismutase (SOD), catalase (CAT), and peroxidase (POD), and contents of proline and malondialdehyde (MDA), in transgenic *Arabidopsis thaliana* with overexpression of *GmNAC3* gene under normal growth condition and drought stress. Data are presented as mean ± standard deviation (*n* = 3). WT, wild type. Symbols “*” and “**” indicate significant differences at *p* values of 0.05 and 0.01, respectively.

Growth Condition	SOD (U/g)	CAT (U/g)	POD (U/g)	Proline (μg/g)	MDA (nmol/g)
Normal					
WT	1159.7 ± 46.3	788.2 ± 31.5	96.3 ± 3.9	21.4 ± 1.1	15.7 ± 0.4
OE-1	2636.0 ± 79.1 *	1013.2 ± 40.5 *	271.3 ± 13.5 **	48.2 ± 2.9 *	10.4 ± 0.6 *
OE-2	2249.0 ± 89.9 *	1051.3 ± 42.1 *	256.7 ± 15.3 **	48.6 ± 1.9 *	10.1 ± 0.4 *
OE-3	2621.1 ± 26.2 *	996.8 ± 29.8 *	286.2 ± 11.5 **	45.0 ± 2.7 *	10.5 ± 0.4 *
Drought (treatment with 6% PEG6000)					
WT	515.8 ± 20.6	938.6 ± 28.2	173.7 ± 6.9	29.6 ± 1.4	12.3 ± 0.5
OE-1	4216.6 ± 126.5 **	1314.0 ± 52.6 *	370.5 ± 11.2 *	73.6 ± 2.9 **	6.7 ± 0.2 *
OE-2	4134.2 ± 124.1 **	1379.5 ± 55.2 *	367.3 ± 18.3 *	74.5 ± 2.9 **	5.9 ± 0.3 *
OE-3	4275.4 ± 85.6 **	1258.0 ± 37.8 *	383.3 ± 19.1 *	73.0 ± 2.9 **	6.8 ± 0.4 *

**Table 3:** Primers and their sequences used in this study. “-F” and “-R” stand for the forward and reverse primers, respectively.

Experiment	Primer	Sequence (5′→3′)
Gene cloning	GmNAC3-F	ATGGCCAAACCAAAAATGC
	GmNAC3-R	TTACTTATCTTGGCTACCACTTCC
Vector construction for subcellular localization	pCAMBIA1302-GmNAC3-F	AGATCTATGGCCAAACCAAAAATGC
	pCAMBIA1302-GmNAC3-R	ACTAGTCTTATCTTGGCTACCACTTCC
Vector construction for transcriptional activation	GmNAC3-pBridge-F	CGTTACTAGTGGATCCATGGCCAAACCAAAAATGC
	GmNAC3-pBridge-R	AGGGAATATTAAGCTTTTACTTATCTTGGCTACCACTTCC
Verification of positive plant materials	35S-F	ACTGGTGATTTCAGCGTGTCC
	35S-R	GCTAGAGCAGCTTGCCAACAT
	Bar-F	TCAAATCTCGGTGACGGGC
	Bar-R	GCACCATCGTCAACCACTACATC
Internal reference gene for qRT-PCR	Tublin-F	GGAAGGCTTTCTTGCATTGGTA
	Tublin-R	AGTGGCATCCTGGTACTGC
*GmNAC* gene expression	qGmNAC3-F	TGACTGGGTCTTGTGTAGGATTTAC
	qGmNAC3-R	GTTCACTGTTATTGTTTGCTGGTG
*APX2* gene expression	APX2-F	CAACCGTGAGCGCTGATTAC
	APX2-R	TCACGTCGTAAGTTCCAGCA
*LEA14* gene expression	LEA14-F	GTATCGTTGGGTGTGATCGGT
	LEA14-R	TAGCCAAGTACTCGACGCTG
*6PGDH* gene expression	6PGDH-F	ACTGATCAACCTGTAGACAAGAAA
	6PGDH-R	GGCCAGTTCACCCAACTTCA
*P5CS* gene expression	P5CS-F	TCACTCGCCAAGATGGAAGG
	P5CS-R	ACTTGCGGCTTCTGAAGGTC

## Data Availability

Not applicable.
